# Identification of circadian rhythm-related genes in colorectal cancer by integrating bioinformatics and multi-omics mendelian randomization

**DOI:** 10.1007/s00210-026-05187-y

**Published:** 2026-03-09

**Authors:** Zhengjie Shen, Minxian Tao, Yin Qin, Wenkai Tang, Yuan Yuan, Wenzhe Gu

**Affiliations:** 1 Department of Oncology, Zhangjiagang TCM Hospital Affiliated to Nanjing University of Chinese Medicine, Zhangjiagang, 215600 China; 2 Department of Orthopaedics, Zhangjiagang TCM Hospital Affiliated to Nanjing University of Chinese Medicine, Zhangjiagang, 215600 China; 3 Department of Rehabilitation, Zhangjiagang TCM Hospital Affiliated to Nanjing University of Chinese Medicine, Zhangjiagang, 215600 China; 4 Department of Otolaryngology, Zhangjiagang TCM Hospital Affiliated to Nanjing University of Chinese Medicine, Zhangjiagang, 215600 China

**Keywords:** Circadian rhythm, Quantitative trait loci, Therapeutic targets, Mendelian randomization, Colocalization, Colorectal cancer, MQTL, EQTL, PQTL

## Abstract

Circadian rhythm disorders are epidemiologically linked to colorectal cancer (CRC), but causal relationships and molecular mechanisms remain unclear. We applied a multi-omics Mendelian Randomization (SMR) framework to assess the causal effects of circadian rhythm-related genes on CRC. Instrumental variables were derived from blood-based QTLs for methylation (mQTL), expression (eQTL), and protein levels (pQTL), with validation using tissue-level eQTLs. Summary data for CRC came from the FinnGen R12 GWAS. Colocalization analyses confirmed shared genetic variants. Candidate genes were further evaluated for expression and prognostic value using TCGA and GEPIA2. Through blood-level SMR analysis, we identified 142 methylation loci, 11 genes, and 2 proteins associated with CRC in discovery cohort. Among them, 42 methylation loci, 3 genes, and 2 proteins were colocalized with CRC incidence. Our SMR analysis highlighted *GRHPR* as a key gene, which is negatively correlated with CRC risk at multiple molecular levels (m/e/pQTL), supported by robust colocalization evidence. Meanwhile, eQTL results showed that high level expression of *UVSSA* was positively associated with CRC risk. QTL results at the tissue level (Colon_Sigmoid and Colon_Transverse) support a causal relationship between the genes *GRHPR* and *UVSSA* and CRC. Crucially, survival analyses revealed that genetically predicted effects of *NAF1* and *ZNF365* on CRC risk were highly consistent with their prognostic values in clinical outcomes (OS and RFS). However, transcriptome expression profiling of the TCGA database CRC cohort revealed no significant differential expression of GRHPR between CRC and normal tissues, though its expression positively correlated with tumor purity. This integrated multi-omics MR analysis identified 12 circadian rhythm-related genes with potential causal relationships to CRC, highlighting *GRHPR* as a risk-reducing factor, and *NAF1* and *ZNF365* as potential dual-purpose biomarkers for both risk stratification and prognosis. The study provides new insights into regulatory mechanisms and signaling pathways, offering novel clues for future mechanistic research and targeted therapies.

**Supplementary Information:**

The online version contains supplementary material available at 10.1007/s00210-026-05187-y.

## Introduction

Colorectal cancer (CRC) is one of the most common cancers and is the second leading cause of cancer deaths (Patel and Dominitz 2024). Epidemiologic results indicate that in 2020 there were 1.9 million new cases and 900,000 deaths globally (Sung et al. 2021). The 5-year survival rate for metastatic CRC (mCRC) is only 14% (Rumpold et al. 2020). Therefore, it is important to identify non-invasive early diagnostic biomarkers and the development of new therapeutic targets for CRC.

Circadian rhythm, as an intrinsic biological clock system, is jointly regulated by endogenous rhythms and external environmental factors (Patke et al. 2020). In mammals, the circadian rhythm is controlled by the Suprachiasmatic Nucleus (SCN) in the hypothalamus, which maintains rhythmic stability through the Transcription-Translation Feedback Loop (TTFL) (Ono et al. 2021). Recently, epidemiological studies have found that shift workers face a 20–30% elevated risk of CRC, which is associated with *PER2* silencing and *NF-κB*/*STAT3* pathway activation in 45% of tumours (Chen et al. 2025). It has been suggested that circadian rhythms may promote the development of several cancers, including CRC, by triggering unfavorable metabolic consequences such as obesity (Miro et al. 2023). Besides, clinical prospective cohort studies suggest that prolonged night shift work may increase the risk of CRC (Barber et al. 2023). Furthermore, a preclinical study shows that circadian disruption can drive lung metastasis in CRC (Liu et al. 2024). This occurs by promoting the buildup of myeloid-derived suppressor cells and impairing CD8⁺ T cell function in the lungs of mice. Notably, although several studies have demonstrated a role for circadian rhythm in CRC, most studies have focused on individual genes or specific circadian rhythm pathways. Therefore, the causal association of circadian rhythm genes with CRC still needs to be supported by more systematic genetic evidence.

Mendelian Randomization (MR), a technique bridging the gap between observational studies and randomized controlled trials (RCTs), is increasingly acknowledged for its ability to bolster causal inferences from non-experimental data (Burgess et al. 2015). By employing genetic variants as instrumental variables (IVs), MR assesses the putative causal relationships between sustained exposures and health outcomes (Davey Smith and Hemani 2014). This approach is advantageous as it circumvents confounding and reverse causality issues. Summary-data-based Mendelian Randomization (SMR) helps assess whether genetically influenced traits, such as gene expression, DNA methylation, or protein levels, are causally linked to complex diseases (Liu et al. 2021). It can also detect potential pleiotropic effects. Furthermore, colocalization analysis is crucial for discerning genuine causal links within the extensive landscape of linkage disequilibrium (LD) across the genome (Qiu et al. 2024). Genome-wide association studies (GWAS) pinpoint genetic associations with traits by analyzing single nucleotide polymorphisms (SNPs) and correlate these findings with gene expression and methylation data (Qiu et al. 2024). However, the exploration of MR to elucidate the potential causal links between circadian rhythm-related genes and CRC risk remains uncharted.

This study aims to explore the possible pathogenic mechanisms of circadian rhythm-related genes in CRC through multi-omics MR methods. By accurately identifying these genetic markers, we aim to pinpoint key circadian rhythm-related genes involved in CRC mechanisms. We also seek to build a multilevel molecular map that tracks changes during disease progression.

## Methods

### Study design

**Fig. 1:**
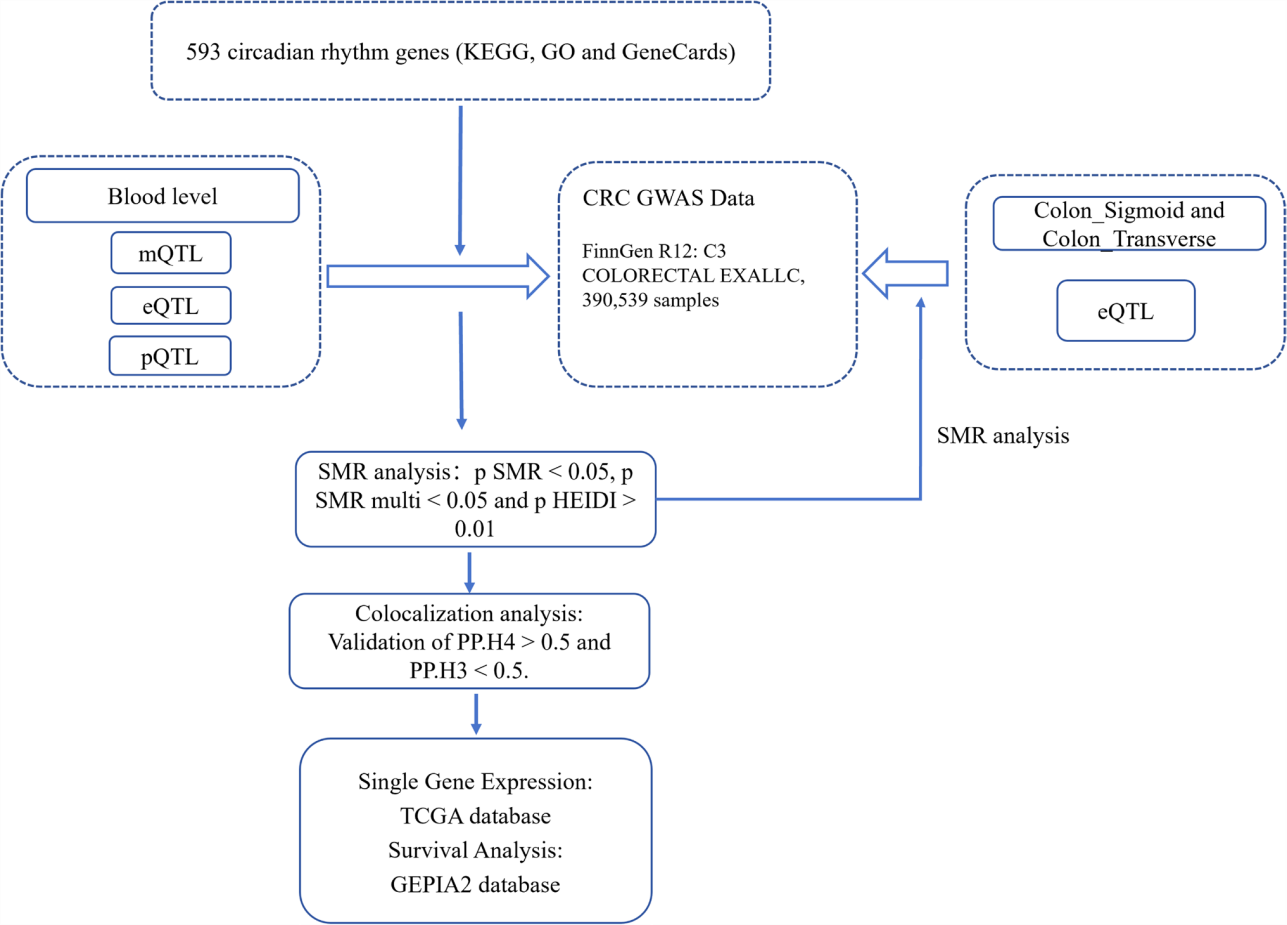
Flowchart of the analyses performed

The CRC dataset used in this study was the FinnGen R12 GWAS database. IVs for circadian rhythm-related genes at methylation, gene expression, and protein abundance levels were extracted from different blood QTL databases. Independent MR analyses were subsequently conducted for CRC at each biological level. Colocalization analyses were then applied to enhance causal inference. By combining the findings from these three distinct levels of SMR analyses, we aimed to pinpoint causal candidate genes. We validated the tissue expression differences and clinical prognostic value of candidate genes using the TCGA and GEPIA2 databases. It is important to mention that all the summary statistics employed in the MR analyses were sourced from publicly accessible studies that had undergone prior ethical review. The study’s design, as well as the process for the selection genetic variants and analytical techniques, is depicted in Fig. 1.

### Data source

We obtained circadian rhythm-related genes in three databases: KEGG (https://www.kegg.jp/entry/ko04710) and GO (Ensembl database, https://www.ensembl.org/Homo_sapiens/Phenotype/Locations?oa=GO:0007623) to extract circadian rhythm-related genes. The GeneCards database (https://www.genecards.org) was searched using the keyword “Circadian rhythm,” and the protein-coding circadian rhythm-related genes were downloaded according to the relevance score > 20. After removing duplicates across three databases, a consolidated set of 593 circadian rhythm-related genes was established (Table S1).

GWAS summary statistics for the trait CRC were extracted from the FinnGen R12 GWAS database (FinnGen R12 C3 COLORECTAL EXALLC) of European ancestry, which included 11,790 cases, 378,749 controls and 20,992,922 SNPs.

Blood mQTL summary data were extracted from a meta-analysis of two European cohorts: the Brisbane Systems Genetics Study (*n* = 614) and the Lothian BirthCohorts (*n* = 1366) (Battle et al. 2017), Blood eQTL summary data came from eQTLGen, involving data from 31,684 individuals (Võsa et al. 2021). Additionally, we extracted genetic summary statistics associated with blood pQTL summary data from Sun et al., which involved 54,219 UK BioBank (UKB) participants (Pietzner et al. xxxx).

Tissue-specific expression of target genes was subsequently assessed by using tissue-specific expression eQTL data retrieved from the GTEx database (https://gtexportal.org/home/) to explore the potential causal impact of genes and CRC. The GTEx v8 dataset included 838 donors and 17,382 samples from 52 tissues and two cell lines. For CRC analyses, we used eQTL data from GTEx Colon Sigmoid and GTEx Colon Transverse.

### SMR analysis

SMR and HEIDI tests were implemented using the SMR software tool (SMR v1.3.1). The SMR analysis method helps us to investigate the relationship between circadian rhythm-related genes (including DNA methylation, gene expression, and protein levels) and the risk of colorectal cancer. The top cis-QTLs were identified within a genomic interval of ± 1000 kb around the gene in question, using a stringent *p*-value cutoff of 5.0 × 10^−8^ to ensure robust detection (Chen et al. 2024). SNPs that demonstrated allele frequency disparities exceeding the predefined threshold (0.2 for this investigation) across any paired datasets, including the linkage disequilibrium (LD) reference panel, QTL summary statistics, and outcome summary statistics, were excluded (Chen et al. 2024). The maximum allowable proportion of SNPs with allele frequency discrepancies was capped at the default threshold of 0.05.

To enhance the statistical efficacy of the study and the reliability of the conclusions, as well as to provide a powerful tool for exploring complex genetic structures, we used a multi-SNP SMR analysis method (SMR-Multi) (Xie et al. 2024). This method considered all SNPs within a certain probe window region of the QTL in the SMR analysis (default value of 500 kb), with *p*-values below the default value of 5 × 10^−8^, and with LD *r*^2^ default value of 0.9 or below for top-related SNPs. Subsequently, the HEIDI test P-HEIDI > 0.01 was used to screen for the absence of pleiotropic results (Chauquet et al. 2021). To avoid the overly conservative nature of strict multiple-testing corrections in multi-omics data, we controlled the false-positive rate through a stringent integrated strategy. Robust causal associations required simultaneously satisfying four criteria: nominal significance in both standard SMR (P_SMR_ < 0.05; P_SMR-Multi_ < 0.05; P_HEIDI_ > 0.01), and independent validation via Bayesian colocalization. For transparency, FDR values are included in the supplementary tables.

In addition to exploring the causal relationship between genes (including DNA methylation, gene expression, and protein levels) and colorectal cancer, the SMR method uses mQTLs (methylation quantitative trait loci) as the exposure and eQTLs (expression quantitative trait loci) as the outcome to further investigate the causal relationship between mQTLs and eQTLs. Similarly, it uses eQTLs as the exposure and pQTLs (protein quantitative trait loci) as the outcome to further explore the causal relationship between eQTLs and pQTLs.

### Colocalization analysis

Colocalization analysis was performed to evaluate whether genetic associations for CRC risk and molecular QTLs (mQTLs, eQTLs, pQTLs) of circadian rhythm genes share the same underlying causal variant at specific genomic loci. The selection of genes was informed by five distinct hypotheses: (1) absence of SNP association with either phenotype (null hypothesis, H0); (2) association with the first phenotype only (H1); (3) association with the second phenotype only (H2); (4) separate variants associated independently with both phenotypes (H3); (5) a common variant influencing both phenotypes (H4). For colocalization analyses of mQTL-GWAS, eQTL-GWAS, and pQTL-GWAS, the window settings for the colocalization region at probe positions were ± 500 kb, ± 1000 kb, and ± 1000 kb, respectively (Battle et al. 2017; Morrow et al. 2018; Yoshiji et al. 2023). QTLs signals satisfying (1) the condition PP.H4 > 0.5 at P12 = 5 × 10^−5^ and (2) the condition PP.H3 < 0.5 at P12 = 1 × 10^−5^ were considered to have successful colocalization with the GWAS signal (Pairo-Castineira et al. 2023).

### Transcriptomic data processing and differential expression analysis

For the TCGA-COAD cohort, we performed differential expression analysis using the DESeq2 package (v1.42.0) in R (v4.3.0). Raw gene-level read counts were used as input. DESeq2 applied its built-in median of ratios method to normalize counts, correcting for differences in library size and sequencing depth. Size factors were estimated using the *estimateSizeFactors()* function, and variance-stabilizing transformation (VST) was applied via the *vst()* function to stabilize dispersion across the mean–variance relationship.

High-throughput transcriptomic sequencing data from 481 primary colorectal adenocarcinoma tumor samples and 41 adjacent normal tissue samples were obtained from the GDC Data Portal. Differential expression analysis was performed using the Wilcoxon rank-sum test via the *wilcox_test* function in the rstatix package (v0.7.2). Significance levels were denoted as *p* < 0.05 (*), *p* < 0.01 (**), and *p* < 0.001 (***).

For analyses involving tumor stage, we included 392 tumor samples with available clinical staging information. To account for tumor microenvironment heterogeneity, we applied the ESTIMATE algorithm (implemented via the tidyestimate R package) to compute Stromal score, Immune score, and ESTIMATE score for each tumor sample. Following the approach by Yoshihara et al. (Yoshihara et al. 2013), we converted ESTIMATE scores into inferred tumor purity using the published formula:

$$Tumor\;purity\:=\:cos(0.6049872018\:+\:0.0001467884\:\times\:ESTIMATE\;score)$$

This inferred tumor purity, reflecting the relative proportion of tumor versus non-tumor (stromal/immune) cells, was used as a continuous covariate in downstream analyses, not as an absolute measurement. We compared tumor purity across clinical stages and evaluated the correlation between *GRHPR* expression and tumor purity using Spearman’s rank correlation.

### Survival analysis of the GEPIA2 database

GEPIA2 is a web server for analysing RNA sequencing expression data from The Cancer Genome Atlas (TCGA) and the Genotype-Tissue Expression Project (GTEx) for the analysis of expression and prognosis of key genes (Tang et al. 2019). Based on the median gene expression levels of CRC (COAD) samples in the TCGA database, patients were divided into two groups and the impact of genes on survival in CRC was assessed using the Kaplan–Meier curve analysis tool. Overall survival (OS) was defined as the time from the initial diagnosis of COAD to the patient’s death or last follow-up. Recurrence-free survival (RFS) was defined as the time from initial diagnosis to disease recurrence. Hazard ratios (HR) with statistical P-values are labelled in the figure.

### Statistical analysis and visualization

Statistical analyses were performed in R (v4.3.0) with the "ggplot2" package utilized for creating Manhattan plots and the "forestplot" package for generating forest plots. The SMRLocusPlot and SMREffectPlot codes were adapted from Zhu et al. (Zhu et al. 2022).

## Results

### MR analyses linking blood molecular QTLs to CRC risk

**Fig. 2:**
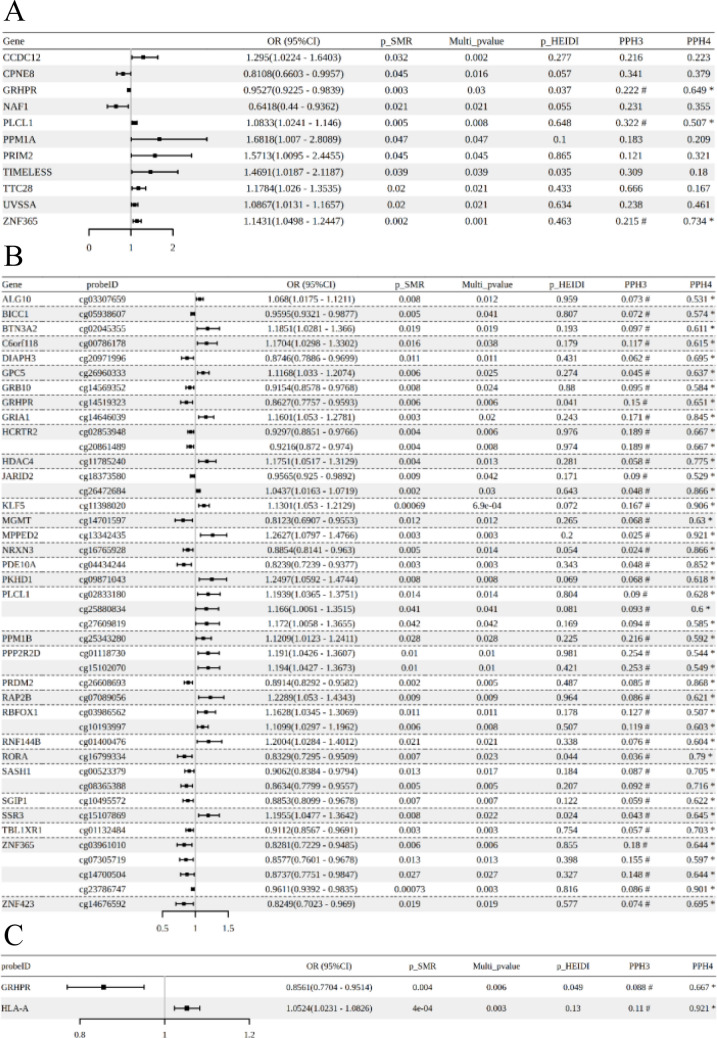
Forest plot results of SMR analysis of circadian rhythm-related gene in FinnGen GWAS cohort. **A** Forest plot of SMR analysis results for eQTLs in the FinnGen R12 cohort. **B** Forest plot of SMR analysis results for mQTLs in the FinnGen R12 cohort. **C** Forest plot of SMR analysis results for pQTLs in the FinnGen R12 cohort

The SMR analysis identified 11 genes that met predefined criteria and were associated with CRC (P-SMR-multi < 0.05, P-SMR < 0.05, and P-HEIDI > 0.01, Fig. 2A). Among these genes, 8 genes were positively associated with an increased susceptibility to CRC (*CCDC12*, *PLCL1*, *PPM1A*, *PRIM2*, *TIMELESS*, *TTC28*, *UVSSA*, and *ZNF365*; odds ratio, OR > 1), while the remaining genes exhibited a negative connection with CRC risk (*CPNE8*, *GRHPR* and *NAF1*, OR < 1). Notably, 3 genes (*GRHPR*, *PLCL1*, and *ZNF365*) showed significant colocalization evidence (PPH4 > 0.5 and PP.H3 < 0.5, Figure S1). The full results of the eQTLs—GWAS analysis of the circadian rhythm genes and CRC are shown in Table S2.

Subsequently, at the DNA methylation levels, SMR analysis identified 142 methylation sites corresponding to 73 genes that met the predefined criteria (P-SMR-multi < 0.05, P-SMR < 0.05, and P-HEIDI > 0.01, Table S3). Among these, 42 methylation sites from 32 genes showed strong colocalization evidence (PP.H4 > 0.5 and PP.H3 < 0.5, Fig. 2B). Meanwhile, there were four overlapping genes identified in both methylation and expression analyses (*PLCL1*, *ZNF365*, *GRHPR*, and *CCDC12*). Eight methylation levels corresponding to three genes (*PLCL1*, *ZNF365* and *GRHPR*) received colocalization support with CRC (Figure S2).

Our results found that protein abundance of GRHPR and HLA-A was associated with CRC risk (Fig. 2C and Table S4, GRHPR (OR = 0.8561, 95%CI (0.7704–0.9514), P-SMR-multi = 0.006, P-SMR = 0.004, and P-HEIDI = 0.049) and HLA-A (OR = 1.0524, 95%CI (1.0231–1.0826), P-SMR-multi = 0.003, P = 4 × 10^−4^ and P-HEIDI = 0.13), and there was strong evidence of colocalization for both (Figure S3). At the same time, we found that the gene GRHPR was the only the only gene associated with disease in both m/e/pQTL and SMR analyses of CRC.

### Multi-omics integration reveals potential causal mechanisms

**Fig. 3:**
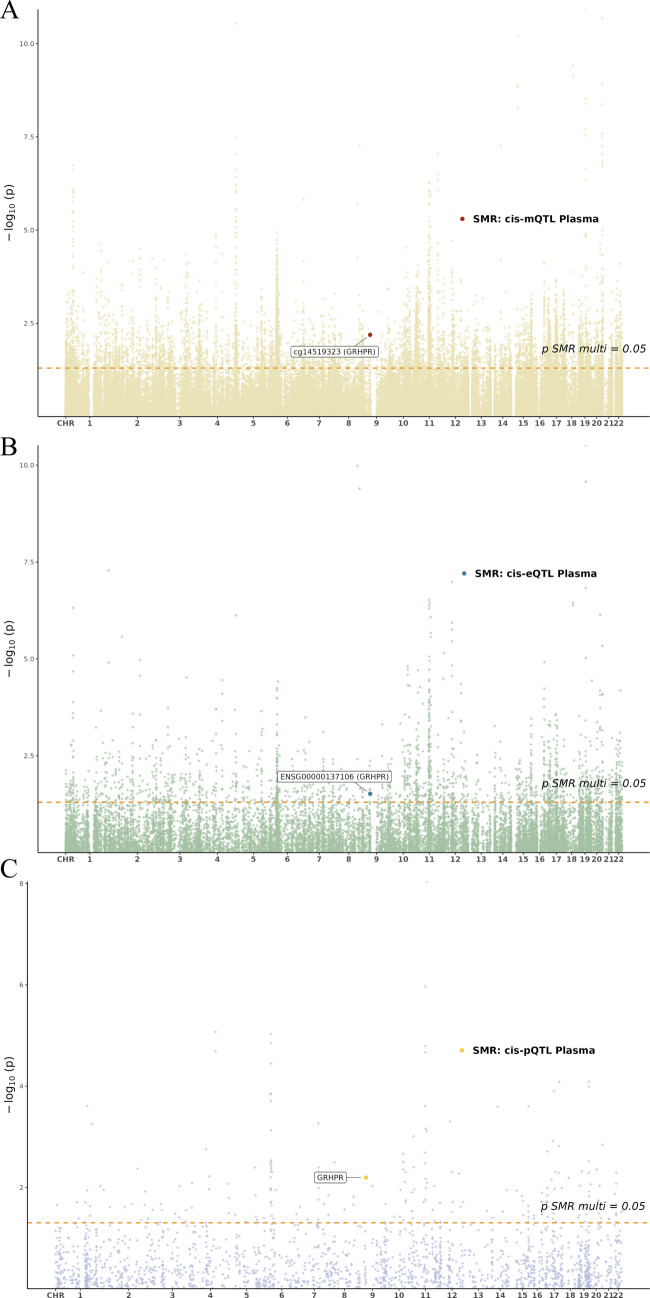
Manhattan plot of FinnGen_R12 cohort mQTLs SMR, eQTLs SMR, and pQTLs SMR analysis results. **A** Distribution of key gene mQTL loci on chromosomes. **B** Distribution of key gene eQTL loci on chromosomes. **C** Distribution of key gene pQTL loci on chromosomes

Manhattan plots of key gene results (gene expression, methylation, and protein abundance) are shown in Fig. 3. Notably, our initial comprehensive SMR analyses identified *GRHPR* as significantly associated with CRC risk across all three molecular levels: methylation, gene expression, and protein abundance (Figure S4). The effect plots in Figure S4A-S4C further illustrate the genetic consistency, or colocalization within a 500 kb window, between the *GRHPR* gene’s mQTL, eQTL, and pQTL GWAS signals and the CRC GWAS signal. Furthermore, the gene *GRHPR* and its corresponding CpG locus were supported by colocalization in all the m/e/pQTL GWAS (Figure S4D-S4F).

To further explore the regulatory relationships among circadian rhythm-related genes at different molecular levels, we first conducted an SMR analysis on the four overlapping genes (*PLCL1*, *ZNF365*, *GRHPR*, *CCDC12*) identified from mQTL and eQTL results. This revealed potential regulatory relationships between 8 methylation sites and these four genes (Table 1 and Table S5). Furthermore, we subsequently performed an SMR analysis using eQTL as the exposure and pQTL as the outcome. The results showed that although *GRHPR* gene expression was positively correlated with its protein level (OR = 1.359, 95%CI (1.333–1.385), P-SMR-multi = 1.0412 × 10^−151^, P-SMR = 3.82 × 10^−223^), this association was strongly rejected by the HEIDI test (P-HEIDI = 1.069 × 10^–11^), indicating that the observed SMR signal is likely driven by linkage disequilibrium rather than a shared causal variant. Despite the complex linkage disequilibrium structures at this specific locus, integrating the robust multi-omics signals led us to hypothesize a hypomethylation/demethylation at the cg14519323 locus suppressed the expression of the *GRHPR* gene, thereby increasing the risk of CRC.

**Table 1:** Results of mQTL—eQTL SMR analysis: possible regulatory relationships

Expo_probe	Outco_Gene	p_SMR	multi_pvalue_SMR	FDR	p_HEIDI	OR (95% CI)
cg19114543	CCDC12	7.1e-17	7.1e-17	2.461E-15	0.021	0.739 (0.688–0.793)
cg14519323	GRHPR	9.7e-16	9.7e-16	3.07E-14	0.171	22.243 (10.432–47.425)
cg02833180	PLCL1	1.7e-10	1.7e-10	3.069E-09	0.186	8.992 (4.582–17.647)
cg25880834	PLCL1	1.9e-09	1.9e-09	2.956E-08	0.336	11.864 (5.293–26.595)
cg27609819	PLCL1	4.4e-09	4.4e-09	6.511E-08	0.352	12.873 (5.483–30.225)
cg03961010	ZNF365	0.006	0.006	0.030797	0.011	0.891 (0.82–0.968)
cg07305719	ZNF365	2.2e-13	2.2e-13	5.608E-12	0.12	0.274 (0.194–0.387)
cg14700504	ZNF365	2.5e-13	2.5e-13	6.274E-12	0.161	0.339 (0.254–0.453)

### Tissue-specific validation results

Further investigation of causal relationships between identified gene expression in tissues and CRC was conducted using tissue types GTEx_Colon_Sigmoid and GTEx_Colon_Transverse. Validation results from GTEx Colon Sigmoid and GTEx Transverse Colon tissues indicate that *GRHPR* gene expression exhibits a negative correlation with CRC risk (All OR < 1), whilst *UVSSA* gene expression demonstrates a positive correlation with CRC risk (All OR > 1; P SMR multi < 0.05, P SMR < 0.05 and P-HEIDI > 0.01; Table 2). Tissue-specific results indicate that 102 and 96 genes associated with CRC were identified in the GTEx sigmoid colon and transverse colon, respectively (Table S6-S7).

**Table 2:** SMR analysis results for FinnGen cohort in GTEx colon sigmoid and colon transverse tissue eQTLs

Data sources	SYMBOL	p_SMR	multi_pvalue_SMR	FDR	p_HEIDI	OR (95% CI)
Colon sigmoid	GRHPR	0.0046666	0.004	0.3219344	0.026	0.9172 (0.86391–0.9738)
Colon sigmoid	UVSSA	0.0191726	0.004	0.4554385	0.626	1.0735 (1.01164–1.13912)
Colon transverse	GRHPR	0.0040586	0.006	0.2870962	0.045	0.9404 (0.90182–0.98066)
Colon transverse	UVSSA	0.0271815	0.022	0.5044128	0.247	1.0631 (1.00691–1.12234)

### TCGA database explores key gene expression

**Fig. 4:**
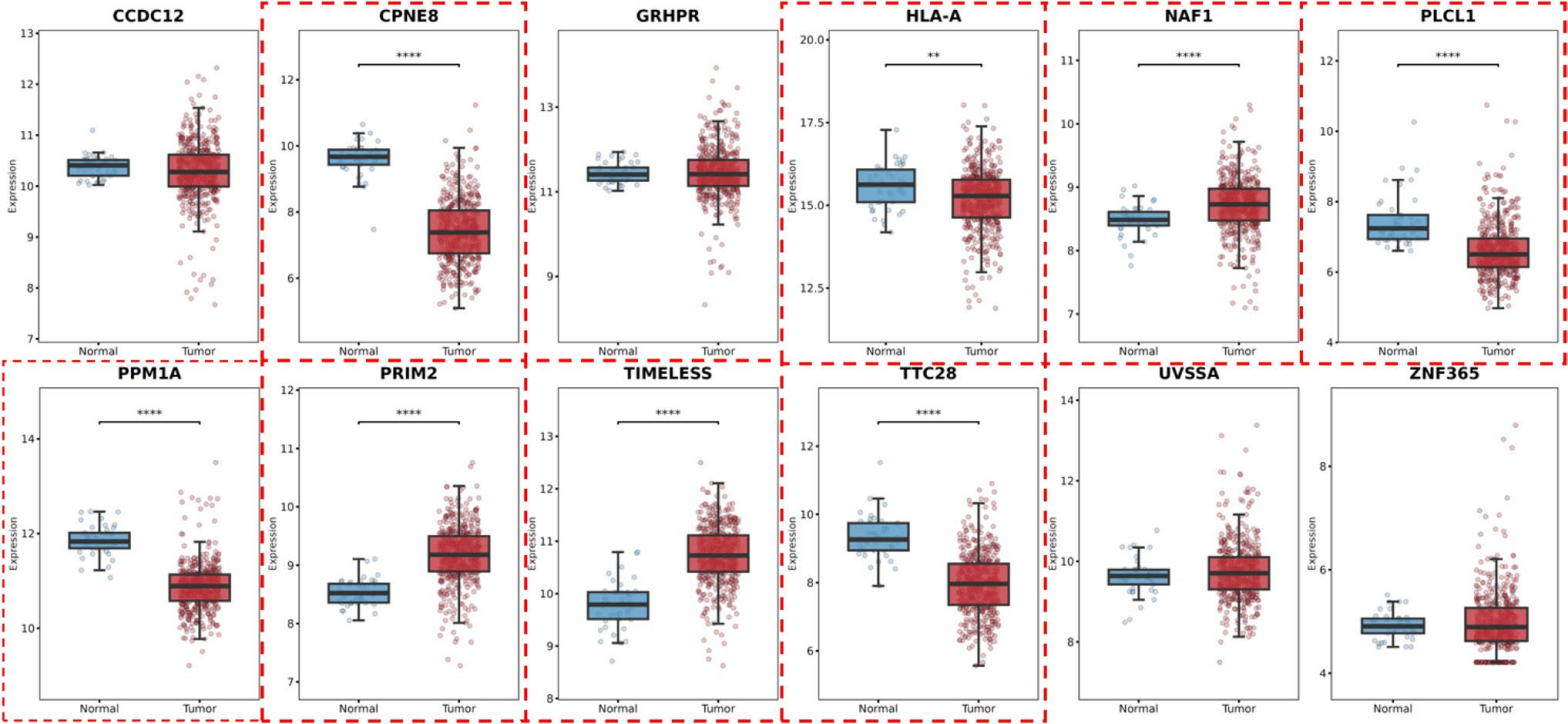
Differential expression of key targets in COAD cancer vs. paracancerous tissues. **A** Gene expression of *CCDC12*. **B** Gene expression of *CPNE8*. **C** Gene expression of *GRHPR*. **D** Gene expression of *HLA-A*. **E** Gene expression of *NAF1*. **F** Gene expression of *PLCL1*. **G** Gene expression of *PPM1A*. **H** Gene expression of *PRIM2*. **I** Gene expression of *TIMELESS*. **J** Gene expression of *TTC28*. **K** Gene expression of *UVSSA*. **L** Gene expression of *ZNF365*

The combined results from the exploratory analysis of eQTL/pQTLs and CRC GWAS identified a total of 12 candidate target genes (*CCDC12*, *CPNE8*, *GRHPR*, *HLA-A*, *NAF1*, *PLCL1*, *PPM1A*, *PRIM2*, *TIMELESS*, *TTC28*, *UVSSA*, and *ZNF365*) exhibiting expression alterations potentially associated with CRC risk. The results showed that the expression of *NAF1*, *PRIM2*, and *TIMELESS* genes was significantly higher in cancer tissues than in peritumoral tissues, whereas the expression of *CPNE8*, *HLA-A*, *PLCL1*, *PPM1A*, and *TTC28* genes was significantly lower in cancer tissues than in peritumoral tissues (Fig. 4). Notably, in the TCGA analysis, *GRHPR* showed no significant differences between tumor and normal groups, nor among different tumor subtypes. Tumor purity distribution exhibited no significant differences across tumor subtypes. *GRHPR* expression was positively correlated with tumor purity (Figure S5).

### The relationship between gene expression levels and survival rates in COAD patients

**Fig. 5:**
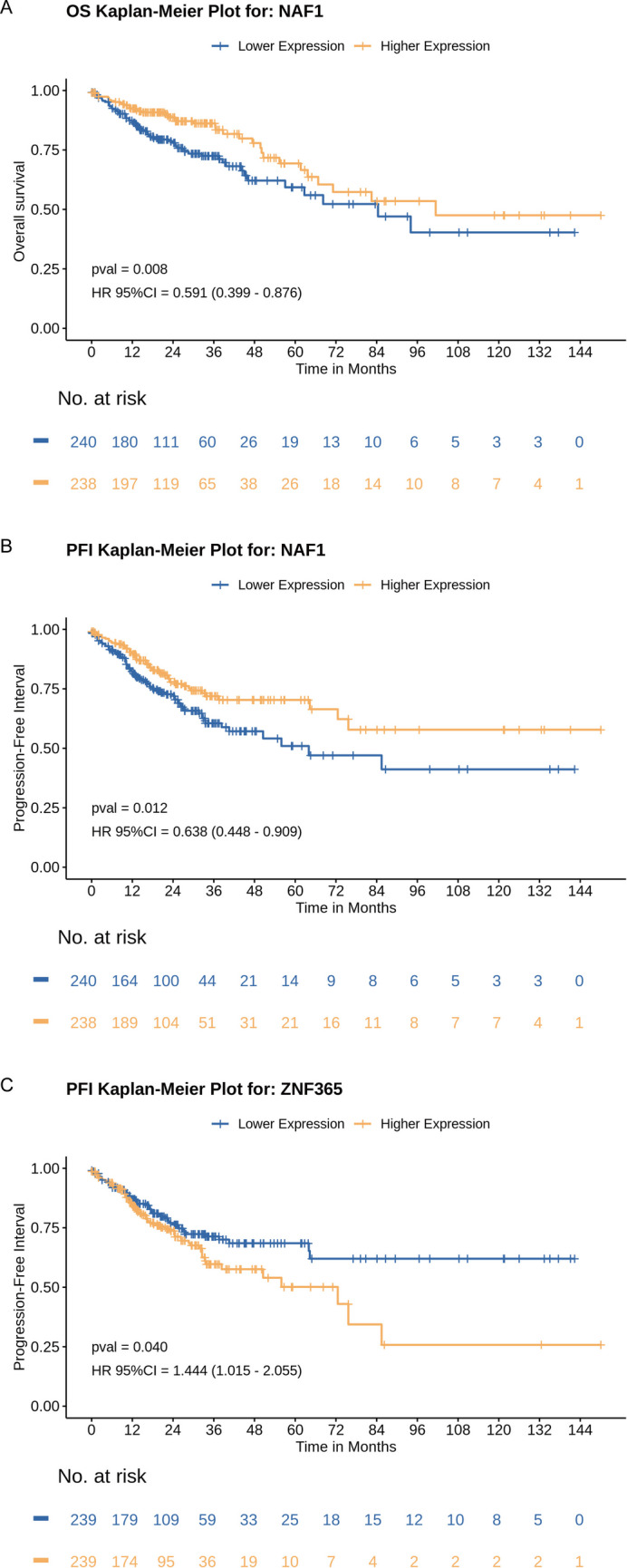
Relationship between gene expression levels and overall or disease-free survival. **A** Relationship between *NAF1* expression levels and overall survival. **B** Relationship between *NAF1* expression levels and disease-free survival. **C** Relationship between *ZNF365* expression levels and disease-free survival

We divided COAD patients into high-expression and low-expression groups based on median gene expression levels for survival analysis. The results showed that, in terms of OS, high *NAF1* expression was significantly associated with better survival rates (*p* < 0.05); in terms of RFS, high *NAF1* expression was linked to better RFS, while high *ZNF365* expression was linked to worse RFS (*p* < 0.05) (Fig. 5).

## Discussion

This study employed a systematic SMR analysis to integrate blood mQTL, eQTL, pQTL, and CRC GWAS data, thereby thoroughly investigating the genetic causal association between circadian rhythm-related genes and CRC risk. We successfully constructed a multi-omics-level evidence chain and identified multiple potential key genes. Among these, *GRHPR* emerged as a novel finding, showing significant and consistent negative associations with CRC risk at the methylation, gene expression, and protein levels, suggesting it may act as a risk-reducing factor in the pathogenesis of CRC.

In this study, the *GRHPR* (glyoxylate/hydroxypyruvate reductase) gene may act as a metabolism-associated gene potentially linked to circadian regulation and exhibit a robust causal association with CRC risk. *GRHPR* and its corresponding protein demonstrated significant causal associations with CRC in SMR analyses across three omics levels: mQTL-GWAS, eQTL-GWAS, and pQTL-GWAS. All analyses passed the HEIDI test (P − HEIDI > 0.01), ruling out the influence of horizontal pleiotropy, thereby greatly enhancing the reliability of the conclusions. Additionally, the causal relationship between *GRHPR* expression and CRC was validated in eQTL data from colorectal tissues (Colon_Sigmoid and Colon_Transverse). The SMR results indicate that genetic variation leads to increased methylation levels at the cg14519323 locus, which in turn upregulates *GRHPR* gene expression, increases GRHPR protein abundance, and ultimately reduces the risk of CRC. Furthermore, SMR results indicate that methylation levels at the cg14519323 site positively correlate with *GRHPR* gene expression. This site is located within the gene body rather than the promoter region (Figure S6) (Jjingo et al. 2012; Xie et al. 2025), suggesting that this CpG site may function as a key epigenetic switch regulating *GRHPR* expression. Consistent with this, elevated cg14519323 methylation correlates with increased *GRHPR* expression and higher protein levels, ultimately conferring reduced CRC risk. Notably, while SMR indicates a significant association, HEIDI testing suggests the potential presence of complex linkage disequilibrium structures. Therefore, the specific molecular transmission mechanism requires further validation. In terms of biological mechanisms, we suggest that *GRHPR* may exert potential CRC risk-reducing effects through metabolic regulation, oxidative stress homeostasis, and interaction with circadian networks. In terms of metabolic regulation, as a key metabolic enzyme, *GRHPR* is mainly involved in the reduction reaction of acetaldehyde and hydroxypyruvate (Katane et al. 2021), and is an important component of the metabolic pathway of glycine, oxalic acid, and hydroxypyruvate in peroxisomes and mitochondria (Wanders et al. 2025). High expression of *GRHPR* can reduce the accumulation of potentially toxic metabolites such as oxalic acid (Martin-Higueras et al. 2019) and prevent DNA damage induced by them or mitochondrial dysfunction, thus reducing the risk of malignant cell transformation. Moreover, by regulating hydroxypyruvate metabolism, *GRHPR* may affect the NAD +/NADH ratio (Lassalle et al. 2016), which may in turn regulate cellular energy status and redox balance, and inhibit preferential metabolic reprogramming (e.g., Warburg effect) in tumour cells. Second, *GRHPR* is involved in reactive oxygen species (ROS)-related metabolism in the peroxisome (Rasekhi et al. 2020). Moderate levels of ROS are essential for cell signaling, but excessive ROS lead to DNA damage and genomic instability, promoting carcinogenesis (Brieger et al. 2012). We suggest that high levels of GRHPR may indirectly reduce ROS production during metabolism or enhance cellular resistance to oxidative damage by maintaining the function of antioxidant systems such as glutathione. Finally, although *GRHPR* is not a core clock gene, it may function downstream of circadian pathways; its expression may be influenced by circadian regulatory networks or, in turn, affect rhythm-related metabolic rhythms. Circadian rhythm disruption has been widely recognized as a risk factor for CRC. Metabolic status is closely linked to cell cycle progression. High levels of GRHPR may indirectly affect the activity of cell cycle regulatory proteins (e.g., p53, p21) by regulating the levels of key metabolites (Hou et al. 2021), promoting DNA damage repair or inducing aberrant apoptosis.

In contrast, differential expression analysis of TCGA-COAD data using a rigorous DESeq2 pipeline revealed no significant difference in GRHPR expression between primary tumor and adjacent normal tissues. This observation does not contradict the SMR findings but rather highlights a key distinction: SMR captures the effect of lifelong, genetically determined expression on disease risk (etiology), whereas TCGA reflects the transcriptional state of established tumors (pathology). The absence of differential expression in late-stage tumors implies that *GRHPR’s* protective influence may be most relevant in pre-malignant or early phases of CRC development, with its expression subsequently normalized or decoupled from disease progression once malignancy is established. Thus, the lack of association in tumor transcriptomes underscores that genetic predisposition and somatic expression changes represent distinct biological layers, one shaping risk, the other reflecting consequence.

In addition, our integrated SMR and survival analyses provide convergent evidence supporting a role in reducing CRC susceptibility for *NAF1* and a detrimental role for *ZNF365* in CRC. SMR results indicate that genetically predicted higher expression of *NAF1* is causally associated with reduced CRC risk (OR = 0.64), while elevated *ZNF365* expression confers increased risk (OR = 1.14). Critically, these genetic associations are mirrored in clinical outcomes: patients with high *NAF1* expression exhibit significantly better OS and longer RFS, whereas high *ZNF365* expression predicts poorer RFS.

These findings suggest that *NAF1* and *ZNF365*, both implicated in circadian regulation and RNA metabolism, may influence CRC not only at the level of disease susceptibility but also in tumor progression and relapse. *NAF1*, a core component of the telomerase complex and H/ACA ribonucleoprotein machinery (Kittur et al. 2006; Stanley et al. xxxx), may promote genomic stability and dampen oncogenic stress, thereby reducing both initiation and recurrence risk. Conversely, *ZNF365*, which regulates DNA damage response and cell cycle checkpoints (Zhang et al. 2013; Urista et al. 2022), might exert context-dependent oncogenic effects when dysregulated, potentially fostering treatment resistance or micrometastatic persistence.

The concordance between causal inference (SMR) and prognostic observation (TCGA survival analysis) strengthens the biological plausibility of these genes as key nodes linking circadian biology to CRC pathogenesis. Together, they highlight the potential of circadian-related genes as dual-purpose biomarkers, for both risk stratification and post-treatment monitoring, and underscore the value of integrating genetic causality with clinical transcriptomics in oncology research.

One strength of our study lies in the integrated use of MR and colocalization methodologies, which leverage genetic variation to estimate the causal effects of circadian rhythm-related genes methylation expression and protein abundance on CRC risk. MR designs and colocalization methods complement each other effectively by mitigating confounding and reverse causation biases, thereby enhancing the precision of causal inferences. Notably, the inclusion of evidence from multiple omics levels in our analysis further bolsters the argument for a causal relationship between circadian rhythm-related genes and the risk of CRC. The substantial sample size of the GWAS dataset used in this study substantially improved its statistical power. The consistency of our findings across various datasets lends additional credibility to our results. Any research methodology has its strengths and, of course, its limitations. Firstly, among the eQTL and mQTL included in the study, there is no information on genetic variation on the X and Y chromosomes. Moreover, despite the multi-omics approach employed in our study, it remains fundamentally observational, thus limiting our ability to establish causation conclusively. Third, our study only includes eQTL and mQTL in the cis-region. Future research endeavors should incorporate functional validation studies to substantiate the implications of our findings. Fourth, our data primarily originates from European populations (FinnGen). Due to genetic and environmental differences, it may not be directly applicable to non-European groups (such as those of Asian or African descent) and requires validation in diverse cohorts. Finally, this study is based primarily on data from European populations, and future studies should further explore gene-disease associations in other ethnicities and populations.

In summary, this study establishes for the first time that the *GRHPR* gene acts as a genetic risk factor for reducing CRC risk; reveals a novel molecular link between circadian rhythms and cancer; and elucidates how gene function dynamically changes during tumorigenesis and progression. Additionally, the identification of NAF1 and ZNF365 as robust predictors for both CRC susceptibility and patient survival highlights their potential as dual-purpose clinical biomarkers. These findings provide an important theoretical basis and potential targets for early risk prediction, molecular typing and targeted intervention (e.g., activation of the GRHPR pathway) in CRC. Future studies should further validate the spatiotemporal-specific roles of these genes in functional experiments and clinical cohorts to promote the development of precision prevention and treatment strategies.

## Supplementary Information

Below is the link to the electronic supplementary material.

ESM1 (JPG 3.45 MB)

ESM2 (JPG 1.99 MB)

ESM3 (JPG 2.44 MB)

ESM4 (JPG 926 KB)

ESM5 (PNG 554 KB)

High Resolution Image (5.17 MB)

ESM6 (PNG 736 KB)

High Resolution Image (6.03 MB)

ESM7 (DOCX 12.6 KB)

ESM8 (XLSX 1.89 MB)

## Data Availability

All data generated or analysed during this study are included in this published article.

## References

[CR1] Barber LE, VoPham T, White LF, Roy HK, Palmer JR, Bertrand KA (2023) Circadian disruption and colorectal cancer incidence in black women. Cancer Epidemiol Biomarkers Prev 32:927–935 36409509 10.1158/1055-9965.EPI-22-0808 PMC10199956

[CR2] Battle A, Brown CD, Engelhardt BE, Montgomery SB (2017) Genetic effects on gene expression across human tissues. Nature 550:204–213 29022597 10.1038/nature24277 PMC5776756

[CR3] Brieger K, Schiavone S, Miller FJ Jr., Krause KH (2012) Reactive oxygen species: from health to disease. Swiss Med Wkly 142:w13659 22903797 10.4414/smw.2012.13659

[CR4] Burgess S, Timpson NJ, Ebrahim S, Davey Smith G (2015) Mendelian randomization: where are we now and where are we going? Int J Epidemiol 44:379–388 26085674 10.1093/ije/dyv108

[CR5] Chauquet S, Zhu Z, O’Donovan MC, Walters JTR, Wray NR, Shah S (2021) Association of antihypertensive drug target genes with psychiatric disorders: a Mendelian randomization study. JAMA Psychiatr 78:623–631 10.1001/jamapsychiatry.2021.0005 PMC7948097 33688928

[CR6] Chen J, Ruan X, Sun Y, Lu S, Hu S, Yuan S et al (2024) Multi-omic insight into the molecular networks of mitochondrial dysfunction in the pathogenesis of inflammatory bowel disease. EBioMedicine 99:104934 38103512 10.1016/j.ebiom.2023.104934 PMC10765009

[CR7] Chen L, Wang Z, Xiao N, Liu J, Tao Y, Zhang S (2025) Deciphering the circadian rhythm in colorectal cancer: a bibliometric analysis of research landscape and trends. Front Oncol 15:1591257 40589644 10.3389/fonc.2025.1591257 PMC12206864

[CR8] Davey Smith G, Hemani G (2014) Mendelian randomization: genetic anchors for causal inference in epidemiological studies. Hum Mol Genet 23:R89-98 25064373 10.1093/hmg/ddu328 PMC4170722

[CR9] Hou Y, Lin J, Wang D, Zhang Y, Liang Q, Chen N et al (2021) The circular RNA circ_GRHPR promotes NSCLC cell proliferation and invasion via interactions with the RNA-binding protein PCBP2. Clin Exp Pharmacol Physiol 48:1171–1181 33987874 10.1111/1440-1681.13523 PMC8362189

[CR10] Jjingo D, Conley AB, Yi SV, Lunyak VV, Jordan IK (2012) On the presence and role of human gene-body DNA methylation. Oncotarget 3:462–474 22577155 10.18632/oncotarget.497 PMC3380580

[CR11] Katane M, Matsuda S, Saitoh Y, Miyamoto T, Sekine M, Sakai-Kato K et al (2021) Glyoxylate reductase/hydroxypyruvate reductase regulates the free d-aspartate level in mammalian cells. J Cell Biochem 122:1639–1652 34289161 10.1002/jcb.30110

[CR12] Kittur N, Darzacq X, Roy S, Singer RH, Meier UT (2006) Dynamic association and localization of human H/ACA RNP proteins. RNA 12:2057–2062 17135485 10.1261/rna.249306 PMC1664726

[CR13] Lassalle L, Engilberge S, Madern D, Vauclare P, Franzetti B, Girard E (2016) New insights into the mechanism of substrates trafficking in glyoxylate/hydroxypyruvate reductases. Sci Rep 6:20629 26865263 10.1038/srep20629 PMC4749974

[CR14] Liu Y, Li B, Ma Y, Huang Y, Ouyang F, Liu Q (2021) Mendelian randomization integrating GWAS, eQTL, and mQTL data identified genes pleiotropically associated with atrial fibrillation. Front Cardiovasc Med 8:745757 34977172 10.3389/fcvm.2021.745757 PMC8719596

[CR15] Liu JL, Xu X, Rixiati Y, Wang CY, Ni HL, Chen WS et al (2024) Dysfunctional circadian clock accelerates cancer metastasis by intestinal microbiota triggering accumulation of myeloid-derived suppressor cells. Cell Metab 36:1320–34.e9 38838643 10.1016/j.cmet.2024.04.019

[CR16] Martin-Higueras C, Ludwig-Portugall I, Hoppe B, Kurts C (2019) Targeting kidney inflammation as a new therapy for primary hyperoxaluria? Nephrol Dial Transplant 34:908–914 30169827 10.1093/ndt/gfy239

[CR17] Miro C, Docimo A, Barrea L, Verde L, Cernea S, Sojat AS et al (2023) “Time” for obesity-related cancer: the role of the circadian rhythm in cancer pathogenesis and treatment. Semin Cancer Biol 91:99–109 36893964 10.1016/j.semcancer.2023.03.003

[CR18] Morrow JD, Glass K, Cho MH, Hersh CP, Pinto-Plata V, Celli B et al (2018) Human lung DNA methylation quantitative trait loci colocalize with chronic obstructive pulmonary disease genome-wide association loci. Am J Respir Crit Care Med 197:1275–1284 29313708 10.1164/rccm.201707-1434OC PMC5955059

[CR19] Ono D, Honma K-i, Honma S (2021) Gabaergic mechanisms in the suprachiasmatic nucleus that influence circadian rhythm. J Neurochem 157:31–41 32198942 10.1111/jnc.15012

[CR20] Pairo-Castineira E, Rawlik K, Bretherick AD, Qi T, Wu Y, Nassiri I et al (2023) GWAS and meta-analysis identifies 49 genetic variants underlying critical COVID-19. Nature 617:764–768 37198478 10.1038/s41586-023-06034-3 PMC10208981

[CR21] Patel SG, Dominitz JA (2024) Screening for colorectal cancer. Ann Intern Med 177:Itc49-itc64 38588547 10.7326/AITC202404160

[CR22] Patke A, Young MW, Axelrod S (2020) Molecular mechanisms and physiological importance of circadian rhythms. Nat Rev Mol Cell Biol 21:67–84 31768006 10.1038/s41580-019-0179-2

[CR23] Pietzner M, Wheeler E, Carrasco-Zanini J, Cortes A, Koprulu M, Wörheide MA et al (2021) Mapping the proteo-genomic convergence of human diseases. Sci 374:eabj1541 10.1126/science.abj1541 PMC9904207 34648354

[CR24] Qiu X, Guo R, Wang Y, Zheng S, Wang B, Gong Y (2024) Mendelian randomization reveals potential causal relationships between cellular senescence-related genes and multiple cancer risks. Commun Biol 7:1069 39215079 10.1038/s42003-024-06755-9 PMC11364673

[CR25] Rasekhi M, Mohammadi-Sangcheshmeh A, Daliri M, Bakhtiarizadeh M, Shariati V, Rahimi M et al (2020) Transcriptional profile of ovine oocytes matured under lipopolysaccharide treatment in vitro. Theriogenology 157:70–78 32805644 10.1016/j.theriogenology.2020.07.034

[CR26] Rumpold H, Niedersüß-Beke D, Heiler C, Falch D, Wundsam HV, Metz-Gercek S et al (2020) Prediction of mortality in metastatic colorectal cancer in a real-life population: a multicenter explorative analysis. BMC Cancer 20:1149 33238958 10.1186/s12885-020-07656-w PMC7691098

[CR27] Stanley SE, Gable DL, Wagner CL, Carlile TM, Hanumanthu VS, Podlevsky JD et al (2016) Loss-of-function mutations in the RNA biogenesis factor NAF1 predispose to pulmonary fibrosis-emphysema. Sci Transl Med 8:351ra107 10.1126/scitranslmed.aaf7837 PMC5351811 27510903

[CR28] Sung H, Ferlay J, Siegel RL, Laversanne M, Soerjomataram I, Jemal A et al (2021) Global Cancer Statistics 2020: GLOBOCAN Estimates of Incidence and Mortality Worldwide for 36 Cancers in 185 Countries. CA Cancer J Clin 71:209–249 33538338 10.3322/caac.21660

[CR29] Tang Z, Kang B, Li C, Chen T, Zhang Z (2019) GEPIA2: an enhanced web server for large-scale expression profiling and interactive analysis. Nucleic Acids Res 47:W556–W560 31114875 10.1093/nar/gkz430 PMC6602440

[CR30] Urista J, Maldonado M, Toscano-Marquez F, Ramírez R, Balderas-Martínez YI, Becerril C et al (2022) Lack of ZNF365 drives senescence and exacerbates experimental lung fibrosis. Cells. 10.3390/cells11182848 36139424 10.3390/cells11182848 PMC9497065

[CR31] Võsa U, Claringbould A, Westra HJ, Bonder MJ, Deelen P, Zeng B et al (2021) Large-scale cis- and trans-eQTL analyses identify thousands of genetic loci and polygenic scores that regulate blood gene expression. Nat Genet 53:1300–1310 34475573 10.1038/s41588-021-00913-z PMC8432599

[CR32] Wanders RJA, Groothoff JW, Deesker LJ, Salido E, Garrelfs SF (2025) Human glyoxylate metabolism revisited: new insights pointing to multi-organ involvement with implications for siRNA-based therapies in primary hyperoxaluria. J Inherit Metab Dis 48:e12817 39582099 10.1002/jimd.12817 PMC11670150

[CR33] Xie J, Ma R, Xu X, Yang M, Yu H, Wan X et al (2024) Identification of genetic association between mitochondrial dysfunction and knee osteoarthritis through integrating multi-omics: a summary data-based Mendelian randomization study. Clin Rheumatol 43:3487–3496 39259428 10.1007/s10067-024-07136-7 PMC11489259

[CR34] Xie S, Hagen D, Becker GM, Davenport KM, Shira KA, Stegemiller MR et al (2025) Analyzing the relationship of RNA and DNA methylation with gene expression. Genome Biol 26:140 40405312 10.1186/s13059-025-03617-3 PMC12101012

[CR35] Yoshihara K, Shahmoradgoli M, Martínez E, Vegesna R, Kim H, Torres-Garcia W et al (2013) Inferring tumour purity and stromal and immune cell admixture from expression data. Nat Commun 4:2612 24113773 10.1038/ncomms3612 PMC3826632

[CR36] Yoshiji S, Butler-Laporte G, Lu T, Willett JDS, Su CY, Nakanishi T et al (2023) Proteome-wide Mendelian randomization implicates nephronectin as an actionable mediator of the effect of obesity on COVID-19 severity. Nat Metab 5:248–264 36805566 10.1038/s42255-023-00742-w PMC9940690

[CR37] Zhang Y, Shin SJ, Liu D, Ivanova E, Foerster F, Ying H et al (2013) ZNF365 promotes stability of fragile sites and telomeres. Cancer Discov 3:798–811 23776040 10.1158/2159-8290.CD-12-0536 PMC3710545

[CR38] Zhu Z, Chen X, Wang C, Cheng LJ (2022) Novel genes/loci validate the small effect size of *ERBB2* in patients with myasthenia gravis. PotNAoS 119:e2207273119 10.1073/pnas.2207273119 PMC9459313 35969801

