# Supplementary figures and images for: Identification of circadian rhythm-related genes in colorectal cancer by integrating bioinformatics and multi-omics mendelian randomization
Source: Naunyn Schmiedebergs Arch Pharmacol. 2026 Mar 9;399(8):12241–53. doi: 10.1007/s00210-026-05187-y (PMC13269306; doi:10.1007/s00210-026-05187-y)

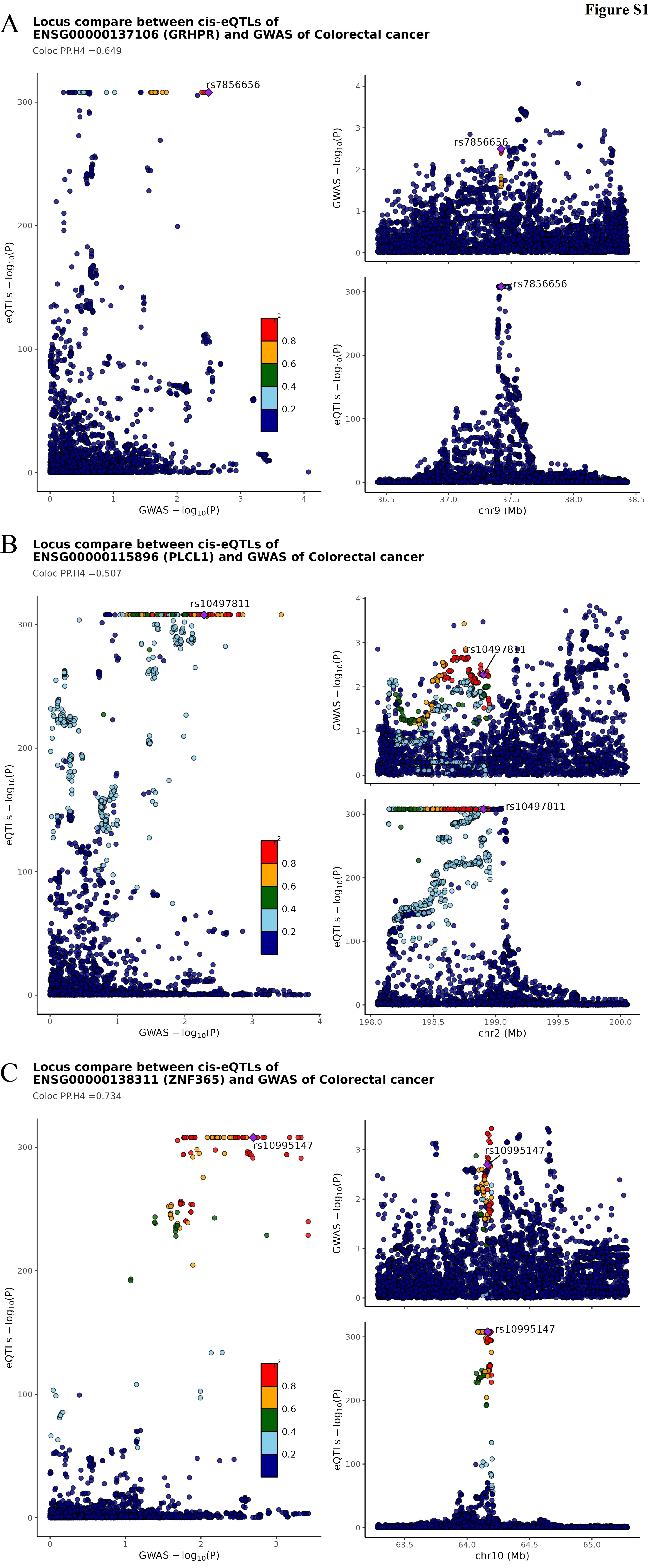

Supplement: Supplementary file 1 — (JPG 3.45 MB) [file 210_2026_5187_MOESM1_ESM.jpg]

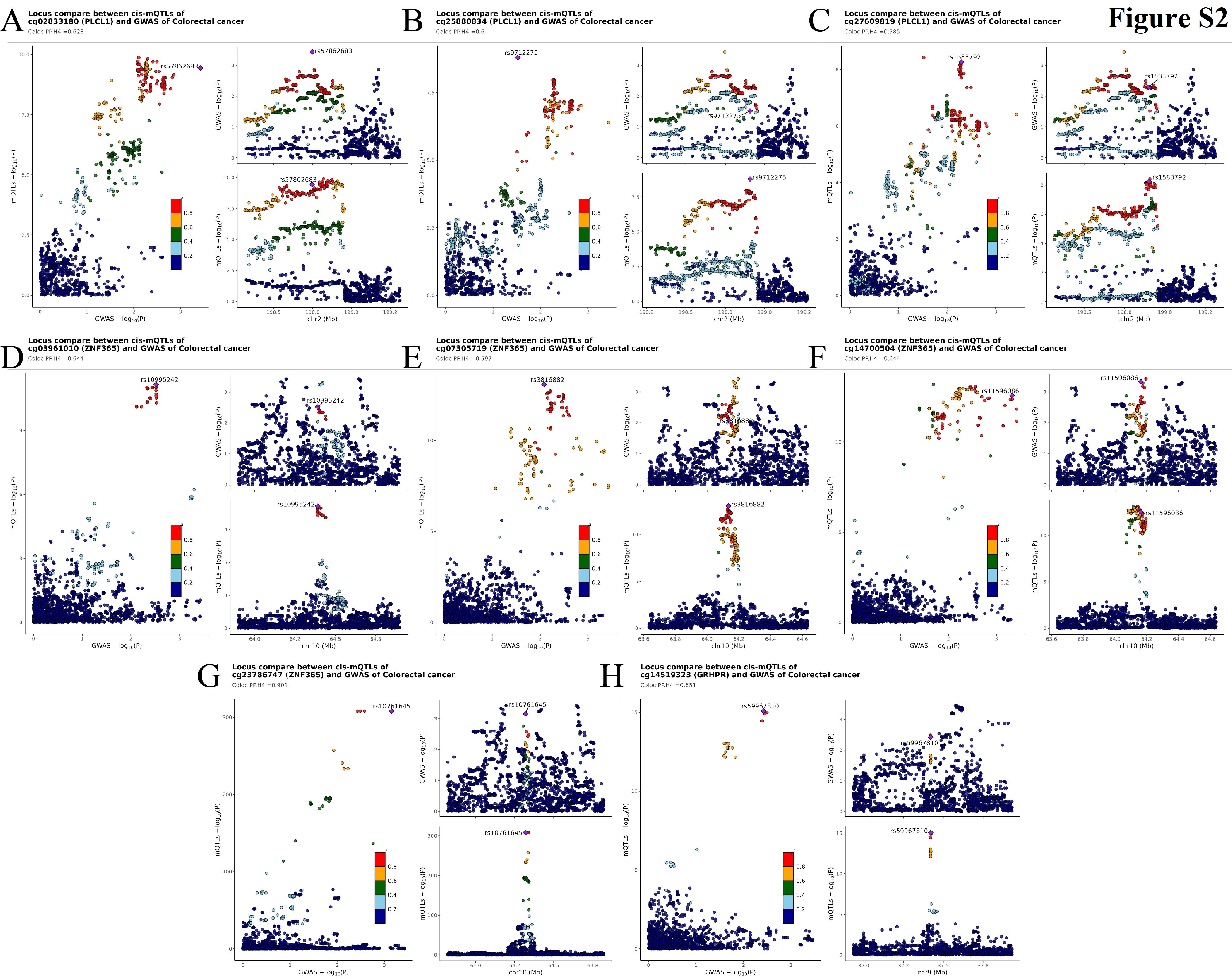

Supplement: Supplementary file 2 — (JPG 1.99 MB) [file 210_2026_5187_MOESM2_ESM.jpg]

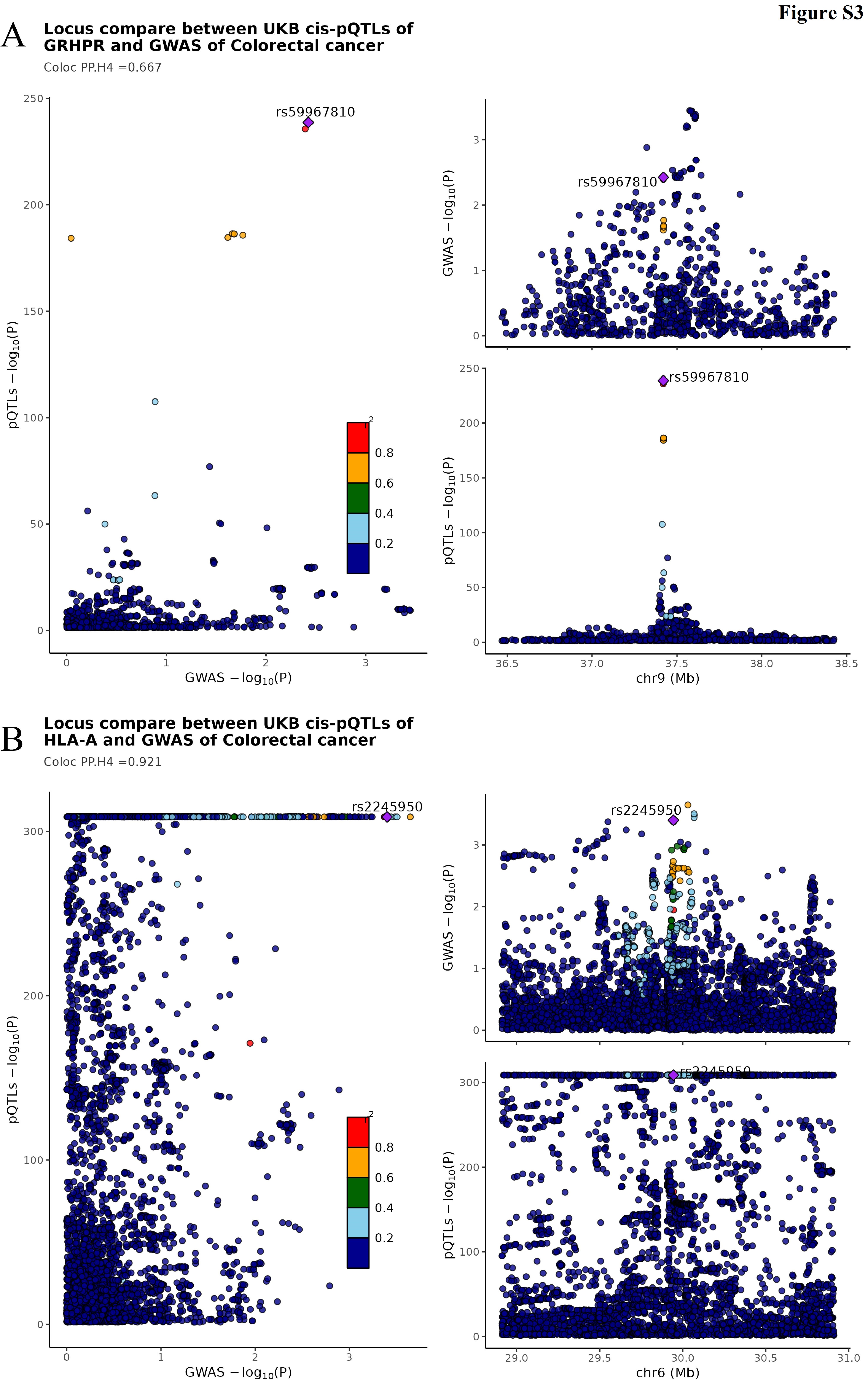

Supplement: Supplementary file 3 — (JPG 2.44 MB) [file 210_2026_5187_MOESM3_ESM.jpg]

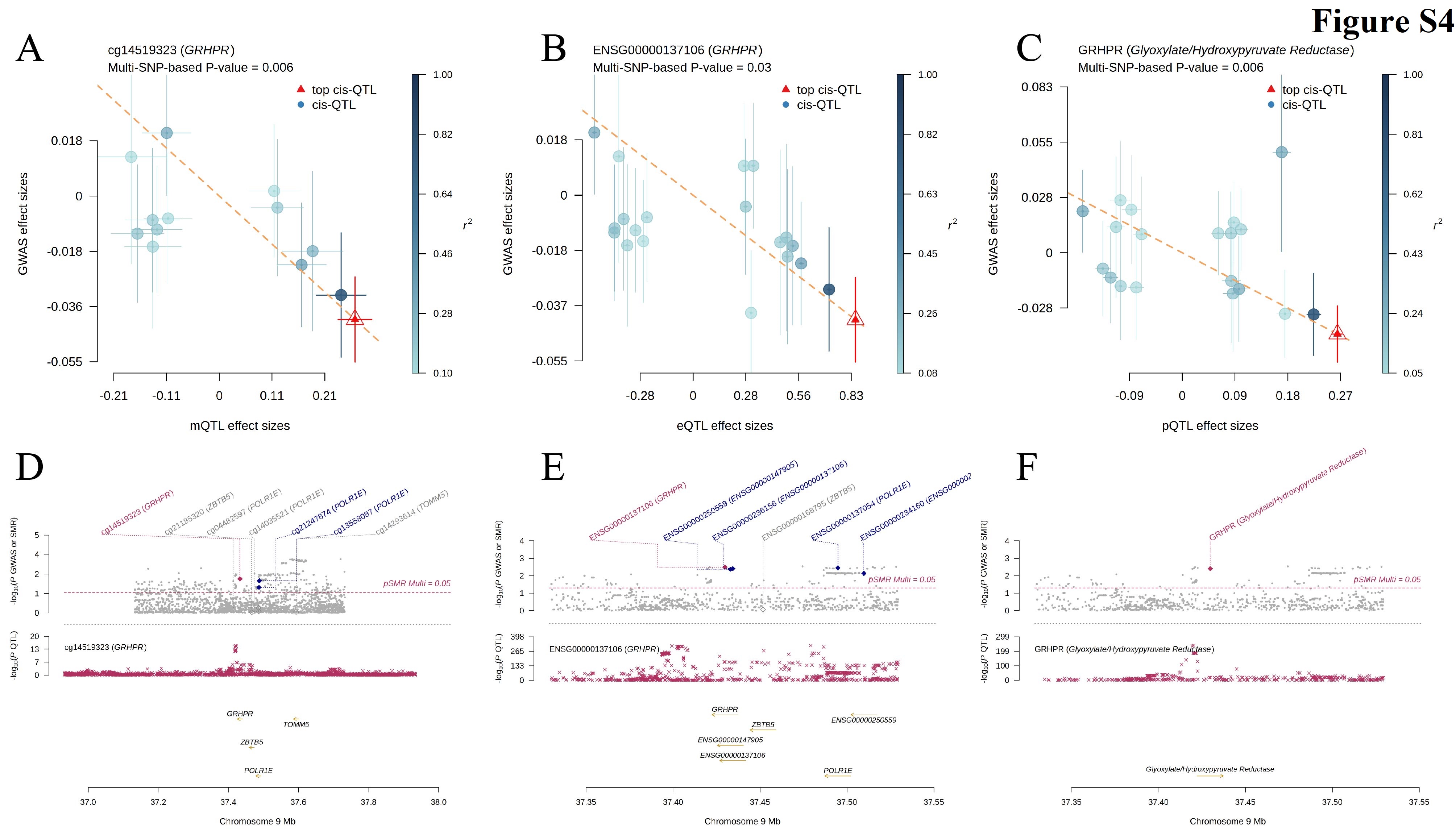

Supplement: Supplementary file 4 — (JPG 926 KB) [file 210_2026_5187_MOESM4_ESM.jpg]

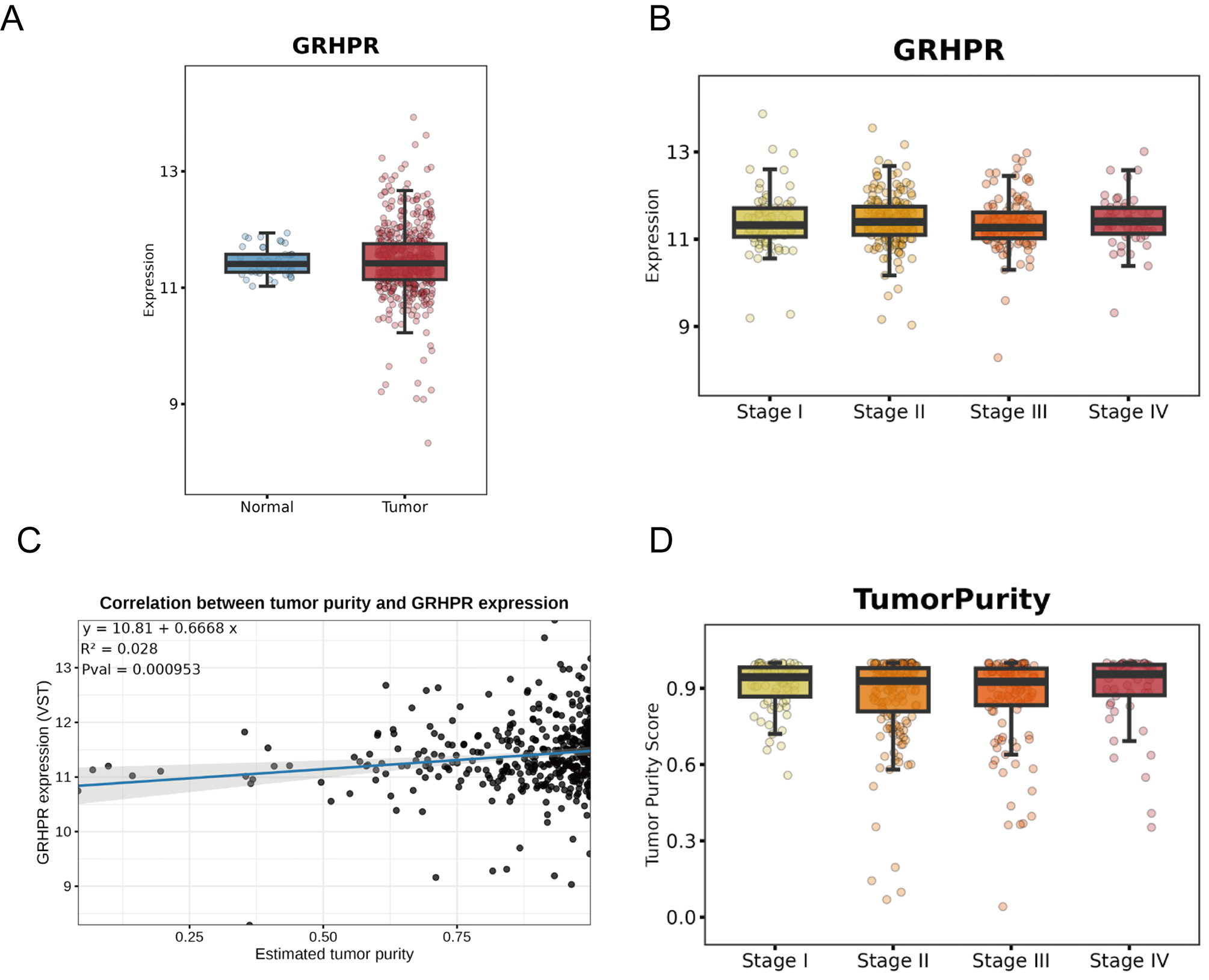

Supplement: Supplementary file 5 — (PNG 554 KB) [file 210_2026_5187_Fig6_ESM.png]

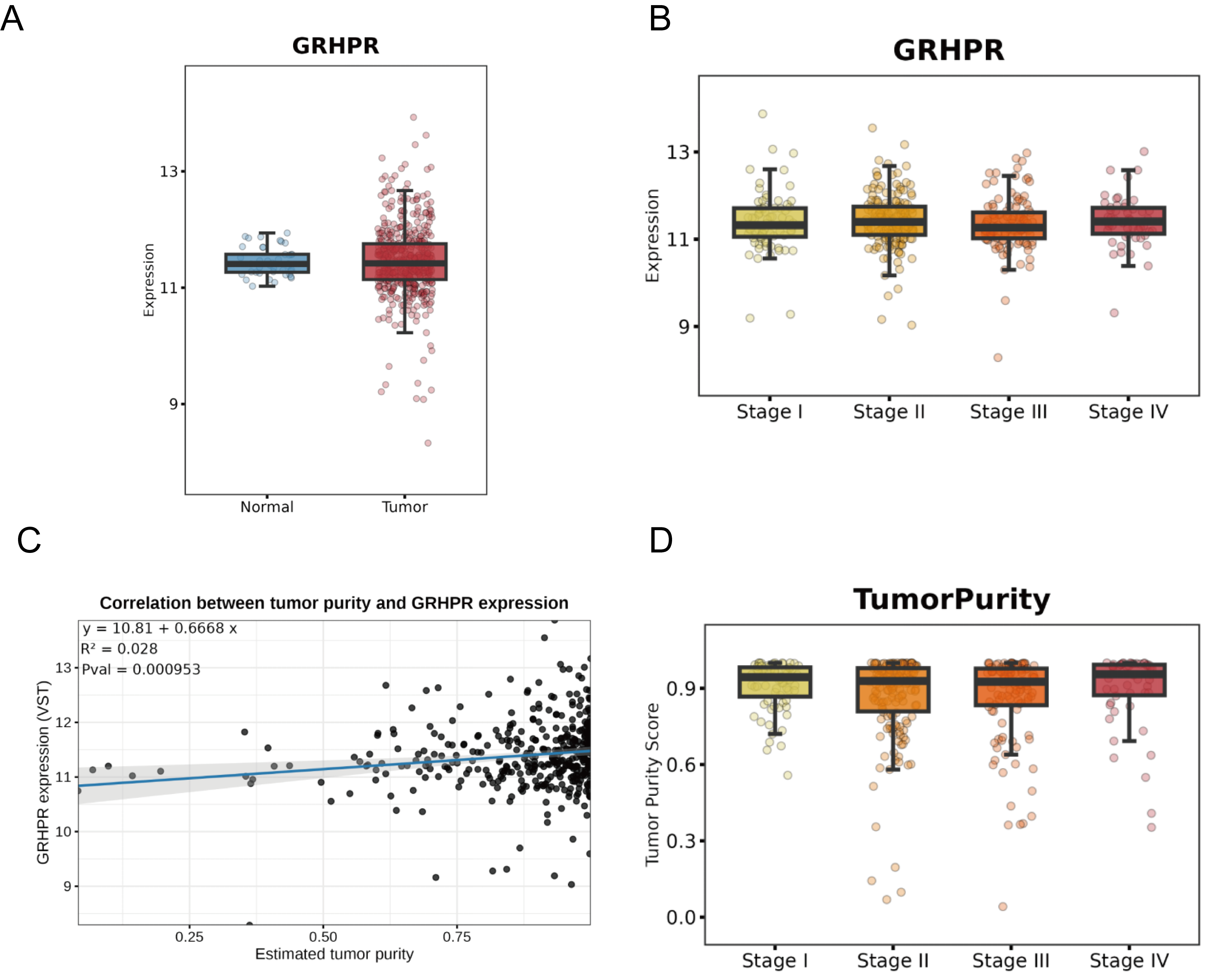

Supplement: Supplementary file 6 — High Resolution Image (5.17 MB) [file 210_2026_5187_MOESM5_ESM.tif]

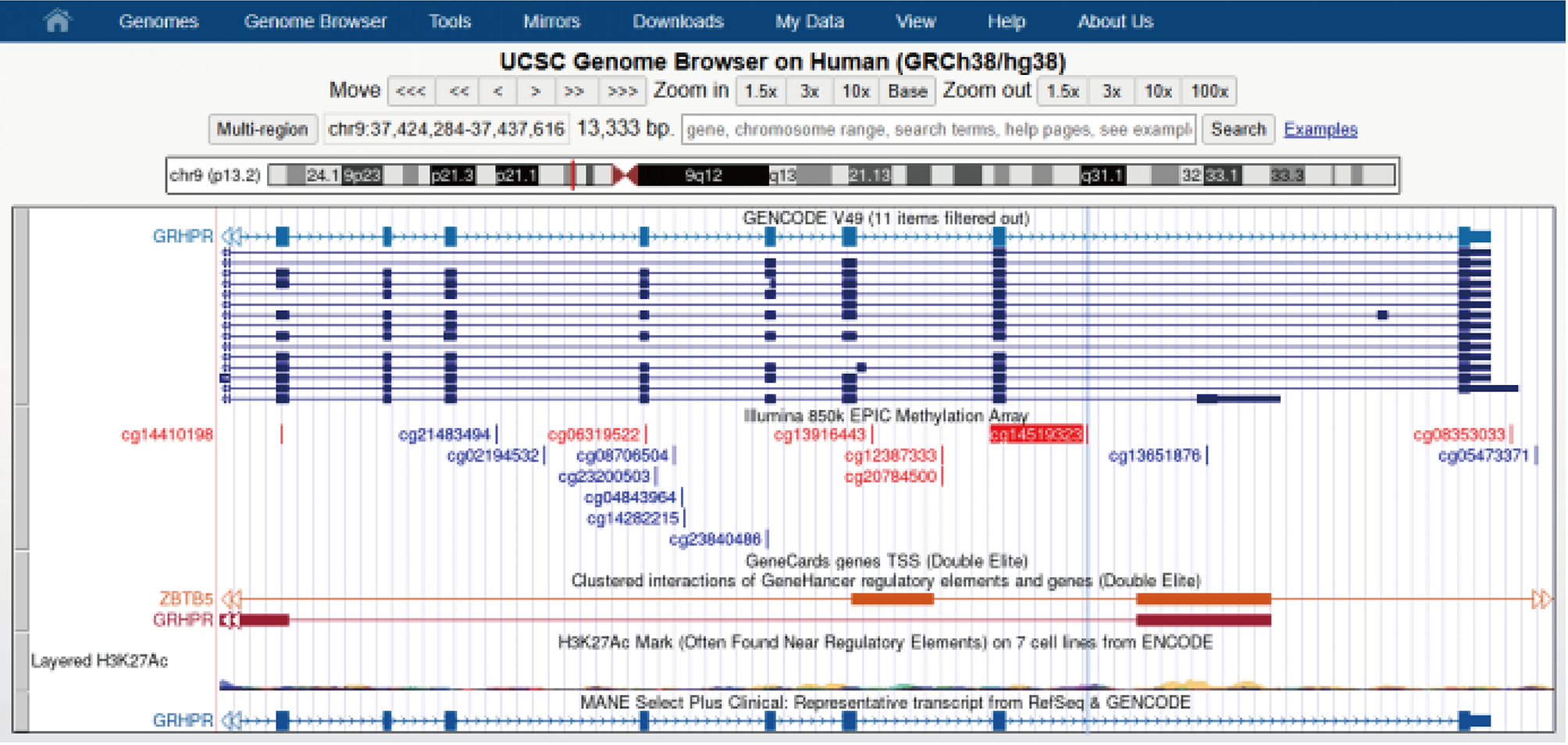

Supplement: Supplementary file 7 — (PNG 736 KB) [file 210_2026_5187_Fig7_ESM.png]

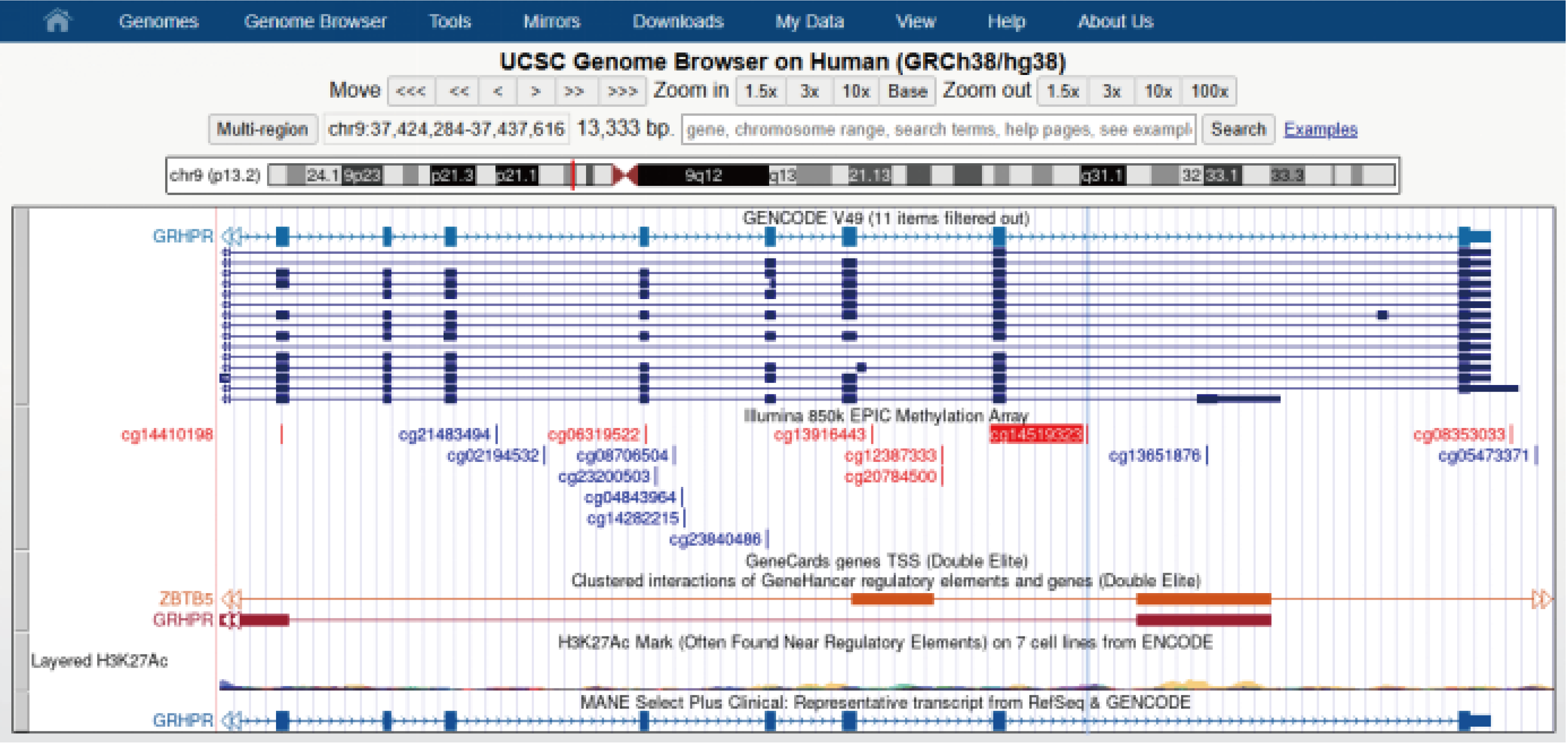

Supplement: Supplementary file 8 — High Resolution Image (6.03 MB) [file 210_2026_5187_MOESM6_ESM.tif]
