# Supplementary material for: Identification of circadian rhythm-related genes in colorectal cancer by integrating bioinformatics and multi-omics mendelian randomization
Source: Naunyn Schmiedebergs Arch Pharmacol. 2026 Mar 9;399(8):12241–53. doi: 10.1007/s00210-026-05187-y (PMC13269306; doi:10.1007/s00210-026-05187-y)
Supplement: Supplementary file 9 — (DOCX 12.6 KB) [file 210_2026_5187_MOESM7_ESM.docx]

**Figure S1. Colocalization results of eQTLs associated with CRC.**

A: *GRHPR*; B: *PLCL1*; C: *ZNF365*.

**Figure S2. Colocalization results of mQTLs associated with CRC.**

A-C: The locus of *PLCL1* in cg02833180, cg25880834 and cg27609819; D-G: The locus fo *ZNF365* in cg03961010, cg07305719, cg14700504 and cg23786747; H: The locus of *GRHPR* in cg14519323.

**Figure S3. Colocalization results of pQTLs associated with CRC.**

A: *GRHPR*; B: *HLA-A*.

**Figure S4. The locus and effect plots of *GRHPR*.**

A: The effect plot of cg14519323 in *GRHPR*; B: The effect plot of *GRHPR* eQTL; C: The effect plot of *GRHPR* pQTL; D: The locus plot of cg14519323 in *GRHPR*; E: The locus plot of *GRHPR* eQTL; F: The locus plot of *GRHPR* pQTL.

**Figure S5. The expression of *GRHPR* in COAD cancer.**

A: The *GRHPR* expression of tissues; B: Expression of *GRHPR* in different stages; C: Correlation between tumor purity and *GRHPR* expression; D: Tumor purity.

**Figure S6. UCSC search reveals that cg14519323 is located within the *GRHPR* gene body.**

**Table S1.** Circadian Rhythm-Related Genes list.

**Table S2.** The SMR results for the association between the expression of Circadian Rhythm-Related Genes and Colorectal Cancer from blood database in discovery cohort (FinnGen cohort).

**Table S3.** SMR analysis from blood DNA methylation to Colorectal Cancer in discovery cohort (FinnGen cohort).

**Table S4.** The SMR results for the association between the protein of Circadian Rhythm-Related Genes and Colorectal Cancer from blood database in discovery cohort (FinnGen cohort).

**Table S5.** The full SMR analysis results for blood mQTLs and eQTLs.

**Table S6.** The SMR results for the association between the expression of Circadian Rhythm-Related Genes and Colorectal Cancer from Tissue Colon Sigmoid.

**Table S7.** The SMR results for the association between the expression of Circadian Rhythm-Related Genes and Colorectal Cancer from Tissue Colon Transverse.
